# Quality analysis of manufacturer's incident reports regarding medical devices

**DOI:** 10.1186/s40545-023-00519-2

**Published:** 2023-01-16

**Authors:** Antonela Simunovic, Krunoslav Kranjcec, Marija Pekas, Siniša Tomic

**Affiliations:** Croatian Agency for Medicinal Products and Medical Devices, Ksaverska Cesta 4, Zagreb, Croatia

**Keywords:** Incident reports, Medical devices, Quality of incident reports, Benefit to risk ratio

## Abstract

**Background:**

Medical devices provide a great number of medical treatments and have an important role in patients’ healthcare; however, there are certain risks, sometimes even serious incidents, associated with their usage. To ensure that benefits of medical device usage always outweigh associated risks, it is necessary to closely monitor known risks post-market and detect new ones as early as possible. Among others, valuable instrument of post-market surveillance is manufacturer incident report. Yet to accomplish its intended use, such report must be sufficiently populated and supplied with correct information. Aim of this paper is to assess the quality of manufacturer’s incident reports received in HALMED since 2012 to May 2021.

**Methods:**

The study included 578 initial reports and 566 final reports that were scored according to the evaluating system we designed and categorized as Excellent, Good, Medium, Qualified and Unqualified. For each report medical device risk class was also extracted to calculate the frequency of report occurrence per risk class and time that passed between the initial and final report. Difference in quality of the reports between manufacturers based on EU countries and countries outside the EU was determined by Mann Whitney *U* test.

**Results:**

Most of initial and final reports fall into two highest quality category level, which means that a sufficient amount of reports are of good/excellent quality and quality of reports prevails. However, the study’s results indicate the need for higher scores, especially in critical fields of the form.

**Conclusions:**

Data obtained from the manufacturer can be scarce and insufficient, causing negative influence on Competent Authority’s investigation procedure. Another issue we recognized is extensive underreporting in Croatia, which can seriously undermine the established system.

## Background

Medical devices continue to gain in importance, offering fast-growing technological advances in the management of a high variety of diseases. However, every medical intervention is associated with expected and unforeseen risks, which may lead to morbidity and mortality of healthcare consumer [1–3].

The EU regulatory environment for medical devices places the responsibility on the manufacturer, or in the case of the manufacturer not having a registered place of business in Europe, their European authorized representative. The manufacturers are responsible for post-market surveillance (PMS) of medical devices, which represents a crucial mechanism to prevent and mitigate potential harm associated with the use of devices. Important part of PMS is vigilance of medical devices [4–9].

European Medical Devices Vigilance System was established in 1995 by the collaboration of the European Commission services, national competent authorities (NCA) and manufacturers with the goal to prevent and reduce the likelihood of recurring incident and to enhance the protection of health and safety of patients, users and others persons [10]. It refers to activities related to collection, assessment, understanding and response to new findings regarding the risks arising from the use or application of medical devices, especially incidents, interactions with other substances or products, contraindications, counterfeiting, deterioration in the characteristics and/or performance of a device, failures and technical malfunctions [11].

Incidents related to medical devices are defined as any malfunction or deterioration in the characteristics or performance of a device made available on the market, including use-error due to ergonomic features, as well as any inadequacy in the information supplied by the manufacturer and any undesirable side-effect [2, 4–6, 11–13].

When incidents meet reporting criteria, manufacturer is obligated to submit an initial incident report to the NCA for recording and evaluation. Every initial report must lead to a final report, unless the nature of incident is such that the initial and the final report can be combined into one report. Users should also report incidents involving medical devices to the manufacturer or to the NCA; however, reporting for users is not legally binding in Croatia [2, 11].

The Croatian Agency for Medicinal Products and Medical Devices (HALMED) implemented the vigilance system in 2008, it includes risk evaluation of incident reports and field safety corrective actions, monitoring of manufacturers' subsequent actions and coordination with other competent authorities when appropriate; however, reporting was initially scarce [2, 11, 14, 15]. During 2012 HALMED focused on education of economic operators and healthcare professionals and set up a more elaborate vigilance management framework.

Manufacturers, economic operators and the national competent authorities are responsible for early identification of effectiveness and risks associated with medical device usage. All parties are obligated to identify risks early and reliably as well as to apply effective and sustainable measures for the safety of patients [9, 10, 16]. PMS system, when correctly designed, should enable a timely identification and communication of potential malfunctions and/or complications related to medical devices that may occur only after years or decades of usage and implement appropriate risk minimization measures [8, 17, 18].

Aim of this paper is to assess the quality of manufacturer’s incident reports received in HALMED since 2012 to May 2021. This analysis is particularly of interest since the new regulatory framework on medical devices strengthens the importance of post-market surveillance and urges EU member states to encourage and enable reporting of suspected serious incidents [3, 8, 12, 13]. As a broader objective, the results of this study are also expected to draw attention to problem of underreporting and insufficient quality of the reports.

## Methods

### Study design

Study included all manufacturer incident reports (MIR) received from year 2012, up to May 2021, at which point the new regulation became applicable. New version of the MIR form, 7.2, was introduced in January of 2020, but became obligatory alongside the new regulation. Study includes only MIR form versions before the 7.2 version.

Total number of reports included in this study is 1144. All reports that were submitted in any form other than MIR form were translated into the corresponding MIR form fields before the assessment.

### Evaluation criteria

HALMED assessors with relevant experience in vigilance reports processing reviewed all MIR form fields to establish which information is considered relevant for proper report interpretation. Fields in the MIR form were divided on fields relevant for report processing, and fields that can be omitted form study. Relevant fields were then assessed and scored with 1 or 2 points based on the content and importance of provided information. Fields containing information crucial for processing of individual incident report and patient risk assessment are considered of higher importance and were appointed with 2 points:

**Table:** 

Manufacturer information	Name
	E-mail
	Country
Authorised Representative Information	Name
	E-mail
Submitter’s information	Name
	E-mail
Medical device information	Commercial name/brand name/make
	Serial number(s) (if applicable)
	Lot/batch number(s) (if applicable)
Incident Information	Date the incident occurred
	Incident description narrative
	Manufacturer’s awareness date
	Medical device current location/disposition (if known)
Patient information	Patient outcome
Healthcare facility information	Name
	E-mail
Results of manufacturers final investigation (Final report)	The manufacturer’s device analysis results
	Is the manufacturer aware of similar incidents with this type of medical device with a similar root cause?
	The medical device has been distributed to the following countries

Wherever possible, free text fields assessed as relevant were evaluated by assessing whether the reporter provided enough information to enable IMDRF coding that is required with the new MIR form. Specifically, Incident description narrative is evaluated for providing enough information for coding Medical device problem codes (Annex A), Patient outcome for coding Health impact (Annex F) and two free text fields in the section Results of manufacturers final investigation (Final report): the manufacturer’s device analysis results and Final comments from the manufacturer were reviewed to provide information for three IMDRF codes: type of investigation (Annex B), investigation findings (Annex C) and investigation conclusion (Annex D).

Other free text fields were assessed only based on criterion- filled, void or incomplete; where incomplete means the field contains some information; however, information is not relevant for that field:

**Table:** 

Patient information	Remedial action taken by the healthcare facility relevant to the care of the patient
Results of manufacturers final investigation (Final report)	Remedial action/corrective action/preventive action/Field Safety Corrective Action
	Time schedule for the implementation of the identified actions
	Further investigations

Fields that were omitted from the study:

- Accessories/associated devices (if applicable) in Medical device information section due to the fact that in most cases it is impossible to know whether the field is void on purpose or by faulty reporting
- Fields contained in section Manufacturer’s preliminary comments (initial/follow-up report) Manufacturer’s preliminary analysis and Initial corrective actions/preventive actions implemented by manufacturer are also omitted, due to lack of a clear criterion for evaluating the information. Practice showed that manufacturers provide very different information in these fields, thus making the decision on information relevance difficult.

Any information provided, but misplaced on the form was considered as lacking. Final scores were translated to percentages then ranked. Report quality was categorized according to the evaluation system’s five levels of classification (total score = 100%): excellent: total incident report quality evaluation score ≥ 90: good: score 80–89; medium: score 70–79; qualified: score 60–69; unqualified: score < 60 points.

### Statistical analysis

The obtained data were scored according to the described evaluation system in EXCEL table. The scores were then used for quality categorization of initial and final reports as Excellent, Good, Medium, Qualified and Unqualified. Total score for each year was divided with number of reports per year to calculate report quality average. Evaluation system also provided insight into which fields have been most and least populated with correct information in majority of the reports and data population practice for critical fields. For each report medical device risk class was also extracted to calculate the frequency of report occurrence per risk class and time that passed between the initial and final report. Difference in quality of the reports between manufacturers based on EU countries and countries outside the EU was determined by Mann Whitney *U* test.

## Results

**Fig. 1:**
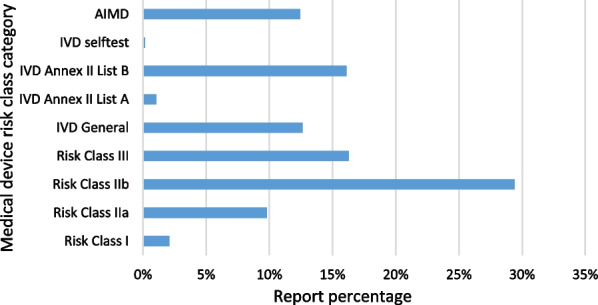
Percentage of each medical device risk class in reports included in the study

The study included 578 initial reports and 566 final reports. Total number of reports per year is available in Table 1, for 2020 and 2021 the number of reports received on MIR form versions previous to 7.2 is indicated in brackets. Percentage of each medical device risk class in reports included in the study is shown in Fig. 1.

**Table 1:** Total number of reports per year

Years	Number of reports
2012	39
2013	22
2014	68
2015	89
2016	68
2017	83
2018	96
2019	108
2020	170 (4)
2021	288 (1)

### Quality of the reports

#### Initial reports

In this study, quality refers to whether the form’s fields are filled with requested information. In total, 143 initial reports (24.74%) reached excellent score, while little more over half, 297 initial reports (51.38%) falls in good score category (Table 2).

**Table 2:** Number of initial reports per category

	Number of reports	%
Excellent	143	24.74
Good	297	51.38
Medium	81	14.01
Qualified	36	6.23
Unqualified	21	3.63

Critical field that was filled the most in initial report is Incident description narrative (99.13%) and the least populated field is Healthcare facility e-mail (43.88%) (Table 3).

**Table 3:** Rate of population of critical fields in initial reports

Field		%
Manufacturer information	Name	98.88
	E-mail	84.69
	Country	97.58
Authorised Representative Information	Name	91.17
	E-mail	90.72
Submitter’s information	Name	96.06
	E-mail	94.71
Medical device information	Commercial name brand name/make	98.10
	Serial number(s) (if applicable)	74.65
	Lot/batch number(s) (if applicable)	71.30
Incident Information	Date the incident occurred	70.50
	Incident description narrative	99.13
	Manufacturer’s awareness date	98.70
	Medical device current location/disposition (if known)	66.26
Patient information	Patient outcome	87.98
Healthcare facility information	Name	83.14
	E-mail	43.88

#### Final reports

In final reports, 335 reports (57.96%) falls in excellent category; however, 16% of the reports are assessed as unqualified (Table 4).

**Table 4:** Number of final reports per category

	Number of reports	%
Excellent	335	57.96
Good	57	9.86
Medium	69	11.94
Qualified	12	2.08
Unqualified	93	16.09

Quality of critical fields is assessed as good, except for information on similar incidents which falls in unqualified category (55.52%), (Table 5).

**Table 5:** Rate of population of critical fields in initial reports

Field		%
Results of manufacturers final investigation (Final report)	The manufacturer’s device analysis results	86.99
	Is the manufacturer aware of similar incidents with this type of medical device with a similar root cause?	55.52
	The medical device has been distributed to the following countries	83.38

#### Time between initial and final report

**Fig. 2:**
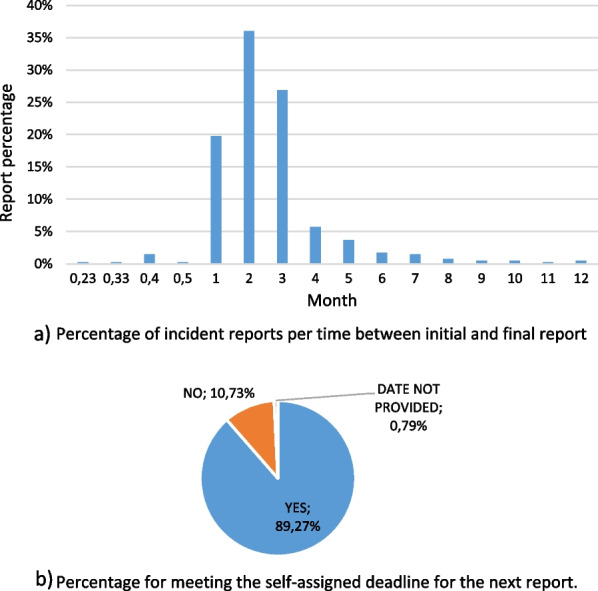
**a** Time passed from initial to the final report; **b** did the manufacturers met the self-assigned deadline

Time passed from initial to the final report was calculated for each report (Fig. 2a) and compared to the field Expected date of manufacturer’s next report to see whether the manufacturers met the self-assigned deadline (Fig. 2b). Most of the incident reports were closed within 2 months and in the majority of the reports, manufacturers followed the self-assigned deadline.

#### Difference in quality of the reports submitted by manufacturers based on EU countries or authorised representative and manufacturers from third countries (Mann Whitney *U* test)

HALMED received 112 initial incident reports and 108 final reports from manufacturers outside EU. Table 6 shows comparison between the reports submitted by manufacturers based on EU countries or authorised representative and manufacturers from third countries.

**Table 6:** Number and percentage of initial and final reports per category

	Number of EU-based initial reports	%	Number of non-EU-based initial reports	%	Number of EU-based final reports	%	Number of non-EU-based final reports	%
Excellent	98	21.03	45	40.18	262	57.21	73	67.59
Good	246	52.79	51	45.54	50	10.92	6	5.56
Medium	71	15.24	10	8.93	62	13.54	7	6.48
Qualified	32	6.87	4	3.57	12	2.62	1	0.93
Unqualified	19	4.08	2	1.79	72	15.72	21	19.44

Median quality of the initial reports from manufacturers based on EU countries and third countries were 84.62% and 87.64%, respectively; the distributions in the two groups differ significantly (Mann–Whitney *U* = 38,598, *n*1 = 466, *n*2 = 112, *p* = 0.0001 two-tailed)

Median quality of the final reports from manufacturers based on EU countries and third countries were 90.91% and 91.67%, respectively; the distributions in the two groups do not differ significantly (Mann–Whitney *U* = 30,191, *n*1 = 458, *n*2 = 108, *p* = 0.7746 two-tailed)

## Discussion

A number of received Manufacturer incident reports shows consistent increase from 2016 to 2021. Even though the application of the REGULATION (EU) 2017/745 (MDR) [12] was postponed, most manufacturers started using the new MIR form in 2020, which is why only four out of 170 MIR reports are included in this study for that year and only one out of 97 for 2021, in the period before the date of the application of the MDR.

Medical devices of every risk class have been represented in the reports; the most reported being risk class IIb medical devices (29.42%). HALMED hosts a national database of medical devices present on the Croatian market as set out in Medical Devices Act (Official Gazette No. 76/13) [11]. We used this database to roughly see different risk class representation on the Croatian market and correlate the information with the number of reports for each risk class. The database shows large representation of 95.273 entries for class IIb. A wide usage of medical devices of certain risk class can influence higher number of reports. Risk class III has similar reporting rate (16.29%) as risk class IVD Annex II List B (16.11%); risk class III has 56.279 entries and class IVD Annex II List B only 635 entries. However, even though the database entry is lower, higher reporting of medical devices classified as IVD Annex II List B may be explained by devices for measuring blood sugar. According to Croatian National Diabetes Registry, the overall number of patients registered in 2020 was 310.212 [19]. Similar reporting rates also show risk classes AIMD (12.43%) and IVD General (12.61%), AIMD having 670 and IVD General 14.417 database entries. Risk class IIa has a reporting rate of 9.81% and 110.958 entries in the database. Risk class I has a rather low reporting rate of 2.1% and 2.915 database entries; however, this low rate can be explained by low risk associated with the usage of medical devices classified in class I. IVD Annex II List A has a reporting rate of 1.05% and 476 entries. The least reported risk class is IVD Devices for self-testing (0.18%) with 105 entries in database. Medical devices for self-testing are used by lay persons and patients who do not have tendency for reporting.

Number of initial report which fall into two highest quality category level is 76.12% (excellent quality 24.74% and good quality 51.38%), which means that a sufficient amount of report still falls into good/excellent category which is indicative for generally good quality of reports.

Out of 17 critical fields, 9 were filled in 90% or higher of report forms showing the adequate level of provided information. Here, we further elaborate critical field that scored lower than 90%. Fields “Manufacturer’s email”, “Patient outcome” and “Healthcare facility name” have a sufficient populating rate of 83.14–87.98%; however, we expect higher rates for those fields since omission of that information cannot be rationally justified. Submitters of reports in these studies are manufacturers, authorized representatives or manufacturer’s subsidiaries, which means the submitters must be familiar with manufacturer’s information, especially the email address. Omitting or incorrectly filling the filed “Patients outcome” significantly hinders the HALMED’s report assessment. The outcome may be unknown at the time of the initial reporting, however, that should be captured in the field. “Healthcare facility name” was omitted in most reports, which delays or completely disables HALMED’s intervention and communication directly with the health care professionals included in the incident. “Serial number(s)” and “Lot/batch number(s)” fields are populated in only 74.65% and 71.30% of reports. This can be explained as in large number of incidents users of medical device do not provide this information what further obstructs the manufacturer’s investigation. However, in those cases, suitable information should be provided in the fields. The field “Medical device current location/disposition” has a lower filling rate of 66.26%, which can also be explained by not knowing this information at the time of initial report, but it should be indicated accordingly. Critical field with lowest information providing rate is “Healthcare facility email” (43.88%), which is concerning since the manufacturer and the Healthcare facility should be in contact during the incident investigation.

Average of initial reports’ quality per year is above 81.98%, except for 2013 (76.98%) and 2019 (79.73%) when it shows a slight decline (Table 7). Average for 2020 and 2021 should not be taken into account due low number of reports taken into account for this study.

**Table 7:** Average of initial reports quality per year

Years	Average (%)
2012	88.06
2013	76.98
2014	83.12
2015	86.72
2016	81.98
2017	83.09
2018	84.52
2019	79.73
2020	67.79
2021	60

Little over half (57.96%) of final reports falls into excellent category of report quality. However, when added to the number of reports in good category it shows that only 67.82% of overall final reporting falls into two highest quality category level. In addition, concerning percentage of reports is assessed as unqualified (16.09%).

Quality of critical fields in final reports “The manufacturer’s device analysis results” (86.99%) and “The medical device has been distributed to the following countries” (83.38%) is assessed as good. However, field “Is the manufacturer aware of similar incidents with this type of medical device with a similar root cause?” scored only 55.52%. Barely more than half of total number of 566 final reports had Information on similar events with the same type of medical device. This information is critical when assessing the incident, it indicates possible broader problem with medical device and potential of causing more incidents. The problem with similar incidents is lack of clear definition and guidelines to determine whether incidents are similar. Manufacturer’s main criterion for claiming similarity is the root cause of the incident; however, any variation of the root cause invalidate similarity between incidents.

Average of final reports’ quality per year is inconsistent trough years. From 2012 to 2019, only 4 years have a good average of quality (above 80%); 2012, 2013 have average assessed as unqualified and 2016 as qualified quality of the reports (Table 8).

**Table 8:** Average of final reports quality per year

Years	Average (%)
2012	54.74
2013	47.01
2014	75.46
2015	81.91
2016	68.67
2017	85.19
2018	91.62
2019	89.95
2020	100
2021	33.33

Time that elapsed in between initial and final reports varied from minimal period of 7 days to the longest period of 12 months. The majority of the manufacturers sent the final reports within 1–3 months after the initial report, the most common period was 2 months (36.05%). In cases where expected date of manufacturer’s next report field was filled in the MIR form, the manufacturer obliged the stated date in 89.27% of cases. Sievanen and Pommelin performed similar quality analysis in Finland in 2003 and observed an increase in average time needed for manufacturer to conclude the investigation of an incident and also that the reports had become more incoherent in terms of the content of information provided in comparison with their previous experience [10].

Comparison of report quality between reporters based on the EU and third countries showed significant difference only for initial reports (*p* = 0.0001). However, it should also be taken into account that initial reports require a greater amount of data, thus it is easier to highlight the difference.

The information provided in the reports is essential for conducting the proper steps of vigilance procedure and for a quick detection of medical device malfunction. Poor quality or insufficient information compromise the evaluation of reports and consequential actions, thus further hampering and preventing the sole purpose of vigilance [20–22].

This study raises questions about the problem of underreporting and possible improvements of the present vigilance system in Croatia. The problem of underreporting is ubiquitous and has been recognised by other competent authorities and countries as well [23]. Main problem of underreporting is not being able to recognize the risk a device presents for the user and the patients. All medical devices have expected risks which are clearly documented in the product information, even without device non-conformities or regulatory non-compliance. Medical device non-conformities can increase likelihood of risk or even introduce new risks related to its usage. Failure to comply with regulations also may be an indicator of a manufacturer’s inability to consistently provide high quality medical devices [24]. The goal of risk assessment related to medical devices is to detect signals as early and reliably as possible to enable considered decisions if a non-acceptable risk is present and, if so, to undertake appropriate measures to mitigate or eliminate the risk [9].

However, one must keep in mind that the number of incidents does not provide enough information by itself about the safety of the device, since a fair percentage of incidents may be caused by unrelated reasons. For example, a big market share would result in more incidents, even though the failure rate is constant or there may be some changes in the incident reporting by the manufacturer [9]. In addition, the safety of a medical device are not only dependent on the product itself and the anatomical and physiological characteristics of the patient, but also very much influenced by the professional or lay user [9, 25].

Besides monitoring the safety of medical devices, another motive for the manufacturers to establish a comprehensive PMS system lies in its innovation potential. PMS system generates information on how medical devices can be designed more efficiently and effectively, resulting in safer and better device usage and risk reduction [26]. Continuous benefit–risk assessment over the device’s life cycle, required under the MDR, will provide an opportunity for the manufacturer to respond to any issue or changes that occur and enable to timely modify the device design or IFU [27].

There is limited number of published information regarding analysis of manufacturers’ incident reports concerning medical devices, especially analysis of the incidents’ quality. New Regulation will set up a centralized database, EUDAMED, which will enable insight in incident reports for all EU countries.

## Conclusion

The study’s results indicate that the data obtained from the manufacturer can be scarce and insufficient, causing negative influence on competent authorities’ investigation procedure. It will be interesting to see if there is any change in the quality of the reports after the implementation of the new Regulations, introduction of the new MIR form and its auxiliary documents. NCAs, including HALMED as the competent authority responsible for vigilance, should provide necessary support and education for manufacturers and authorized representatives to generate high quality incident reports. Another issue we noticed is an extensive underreporting in Croatia, which must be addressed by HALMED through education of healthcare professionals and targeted information campaigns, which should be supported by the national healthcare system and other relevant stakeholders.

## Data Availability

The data set supporting the conclusions of this article is included within the article (and its additional file).

## Abbreviations

PMS: Post-market surveillance
NCA: National competent authorities
HALMED: Croatian Agency for Medicinal Products and Medical Devices
MIR: Manufacturer incident reports

## References

[1] Kramer DB, Tan YT, Sato C, Kesselheim AS (2014). Ensuring medical device effectiveness and safety: a cross-national comparison of approaches to regulation. Food Drug Law J.

[2] EUROPEAN COMMISSION. MEDDEV 2 12–1 rev. 8 Vigilance. European Commission. 2013;(July 2013):1–64. Available at: https://ec.europa.eu/docsroom/documents/32305/attachments/1/translations. Accessed 14 Oct 2021.

[3] Zippel C, Bohnet-Joschko S (2017). Post market surveillance in the german medical device sector—current state and future perspectives. Health Policy.

[4] European Parliament and of the Council. Directive 98/79/EC of the European Parliament and of the Council of 27 October 1998 on in vitro diagnostic medical devices. Official Journal of the European Communities. (October 1998):1–42. https://eur-lex.europa.eu/legal-content/EN/ALL/?uri=celex%3A31998L0079. Accessed 14 Oct 2021.

[5] European Parliament and of the Council . Council Directive 90/385/EEC of 20 June 1990 on the approximation of the laws of the Member States relating to active implantable medical devices. Official Journal of the European Communities. (September 2000):1–15. https://eur-lex.europa.eu/legal-content/EN/ALL/?uri=celex:31990L0385. Accessed 14 Oct 2021.

[6] European Parliament and of the Council. Council Directive 93/42/EEC of 14 June 1993 concerning medical devices. Official Journal of the European Union. (June 1993):1–60. https://eur-lex.europa.eu/legal-content/EN/TXT/?uri=celex%3A31993L0042. Accessed 14 Oct 2021.

[7] Polisena J, Gagliardi A, Urbach D, Clifford T, Fiander M (2015). Factors that influence the recognition, reporting and resolution of incidents related to medical devices and other healthcare technologies: a systematic review. Syst Rev.

[8] Pane J, Francisca RDC, Verhamme KMC, Orozco M, Viroux H, Rebollo I (2019). EU postmarket surveillance plans for medical devices. Pharmacoepidemiol Drug Saf.

[9] Seidel R, Stößlein E, Lauer W (2016). Incident reporting to BfArM—regulatory framework, results and challenges. Biomed Tech.

[10] Sievänen H, Pommelin P (2003). Quality analysis of medical device vigilance reports. Technol Health Care.

[11] Public law: Medical Devices Act (Official Gazette No. 76/13) 18 June 2013.

[12] Regulation (EU) 2017/745 of the European Parliament and of the Council of 5 April 2017 on medical devices, amending Directive 2001/83/EC, Regulation (EC) No 178/2002 and Regulation (EC) No 1223/2009 and repealing Council Directives 90/385/EEC and 93/42/EEC [displayed 14 August 2022]. https://eur-lex.europa.eu/legal-content/EN/TXT/?uri=CELEX%3A32017R0745. Accessed 14 Oct 2021.

[13] Regulation (EU) 2017/746 of the European Parliament and of the Council of 5 April 2017 on in vitro diagnostic medical devices and repealing Directive 98/79/EC and Commission Decision 2010/227/EU [displayed 14 August 2022]. https://eur-lex.europa.eu/legal-content/EN/TXT/?uri=uriserv%3AOJ.L_.2017.117.01.0176.01.ENG&toc=OJ%3AL%3A2017%3A117%3ATOC. Accessed 14 Oct 2021.

[14] Tomic S, Filipovic Sucic A, Plazonic A, Truban Žulj R, Macolic Šarinic V, Cudina B (2010). Regulating medicines in croatia: five-year experience of agency for medicinal products and medical devices. Croat Med J.

[15] The Ministry of Health. Ordinance on Monitoring Adverse Incidents Related to Medical Device (Official Gazette No. 125/13). https://www.halmed.hr/en/O-HALMED-u/Zakoni-i-pravilnici/. Accessed 13 Oct 2021.

[16] Yaqoob T, Abbas H, Shafqat N (2020). Integrated security, safety, and privacy risk assessment framework for medical devices. IEEE J Biomed Health Inform.

[17] Miclăuş T, Valla V, Koukoura A, Nielsen AA, Dahlerup B, Tsianos GI (2020). Impact of design on medical device safety. Ther Innov Regul Sci.

[18] Parvizi N, Robertson I, McWilliams RG (2014). Medical device adverse incident reporting in interventional radiology. Clin Radiol.

[19] Croatia’s Health Insurance Fund. Croatian National Diabetes Registry CroDiab. https://www.hzjz.hr/wp-content/uploads/2021/05/Izvje%C5%A1%C4%87e-za-2020.-godinu.pdf. Accessed 20 Oct 2021.

[20] Ribeiro A, Lima S, Zampieri ME, Peinado M, Figueras A (2017). Filling quality of the reports of adverse drug reactions received at the Pharmacovigilance Centre of São Paulo (Brazil): missing information hinders the analysis of suspected associations. Expert Opin Drug Saf.

[21] Tracol P (2016). Materials vigilance and traceability. Orthop Traumatol Surg Res.

[22] Lassale B, Ragni J, Succamiele L (2018). Computerization of health warnings and incident reports for Materials Vigilance in the Marseille Public Hospitals. Transfus Clin Biol.

[23] Palojoki S, Saranto K, Lehtonen L (2019). Reporting medical device safety incidents to regulatory authorities: an analysis and classification of technology-induced errors. Health Inform J.

[24] U.S. Food and Drug Administration. Factors to Consider Regarding Benefit- Risk in Medical Device Product Availability , Compliance , and Draft Guidance for Industry and. 1500065. 2016;1–32. https://www.fda.gov/regulatory-information/search-fda-guidance-documents/factors-consider-regarding-benefit-risk-medical-device-product-availability-compliance-and. Accessed 12 Jan 2022.

[25] Lange K, Nowak M, Neudörfl C, Lauer W (2017). Umgang mit Patientenmonitoren und ihren Alarmen: Vorkommnismeldungen liefern Hinweise auf Probleme mit Gerätewissen. Zeitschrift fur Evidenz, Fortbildung und Qualitat im Gesundheitswesen.

[26] Zippel C, Bohnet-Joschko S (2017). Innovation for safe and effective medical devices: contributions from postmarket surveillance. Ther Innov Regul Sci.

[27] Wilkinson B, van Boxtel R (2020). The medical device regulation of the European Union intensifies focus on clinical benefits of devices. Ther Innov Regul Sci.

